# Human Myiasis Resulting from Reemergence of *Cochliomyia hominivorax* Screwworm, Mexico, 2025–2026

**DOI:** 10.3201/eid3210.261235

**Published:** 2026-10

**Authors:** Oscar Manuel Delgado-Cuellar, Clemente Mosso-González, Jorge Castañeda-Gómez, Farah Zamira Vera-Maloof, Karla Reyna Navarro-Fuentes, Edgar Sebastián Mejía-Navarro, Héctor Armando Rincón-León

**Affiliations:** Instituto Mexicano del Seguro Social, Unidad de Medicina Familiar No. 1, Tapachula, Mexico (O.M. Delgado-Cuellar); SECIHTI–Instituto Nacional de Salud Pública, Centro Regional de Investigación en Salud Pública, Tapachula (C. Mosso-González, F.Z. Vera-Maloof); Unidad de Investigación en Enfermedades Infecciosas, Cancun, Mexico (J. Castañeda-Gómez); Instituto Mexicano del Seguro Social, Unidad de Medicina Familiar No. 11, Tapachula (K.R. Navarro-Fuentes); Hospital General “Dra. Matilde Petra Montoya Lafragua,” Instituto de Seguridad y Servicios Sociales de los Trabajadores del Estado, Mexico City, Mexico (E.S. Mejía-Navarro); Instituto Mexicano del Seguro Social, OOAD Estatal en Chiapas, Jefatura de Servicios de Prestaciones Médicas, Coordinación Auxiliar Médica de Investigación, Tapachula (H.A. Rincón-León).

**Keywords:** New World screwworm, Cochliomyia hominivorax, myiasis, Mexico, epidemiologic surveillance, One Health, zoonoses, parasites, public health

## Abstract

After its elimination from Mexico in 2003, myiasis caused by New World screwworm (NWS), *Cochliomyia hominivorax*, has recently reemerged as a public and animal health concern. We conducted a retrospective study of human New World screwworm myiasis cases reported through Mexico’s national surveillance system during April 13, 2025–March 14, 2026. Of 204 cases in official surveillance summaries, 202 had sufficient information for case-level clinical and epidemiologic analysis. Cases were concentrated in southern Mexico, particularly Chiapas, followed by Yucatán, Oaxaca, and Quintana Roo. Most (71.8%) cases occurred in men; mean patient age was 61.1 years. Lower limbs (55.0%) were the most frequently reported anatomic location, and 76.7% of patients had >1 concurrent condition. Six deaths were reported among patients with NWS myiasis; at the time of reporting, 1 death was attributed directly to NWS myiasis. Our findings support proactive clinical detection and integrated One Health surveillance along the expected northward spread of NWS.

*Cochliomyia hominivorax*, the New World screwworm (NWS) fly, is an obligate parasite during its larval stage. Adult female flies are attracted to blood, wounds, inflamed tissue, mucous membranes, and natural body orifices, where they deposit eggs that hatch within 12–24 hours. The larvae invade and feed on living tissue for 5–7 days before dropping to the soil to continue development (*1**–**3*). Because female NWS flies usually mate only once, mass release of irradiated sterile flies can interrupt reproduction. That sterile insect technique, combined with quarantine and treatment of infested animals, supported eradications of NWS from North America southward to the Darién Gap in Panama (*4*). After eradication north of the Darién Gap, continued releases of sterile flies were used to maintain a biologic barrier intended to prevent reinvasion from NWS-endemic areas of South America. Environmental conditions, including moderate-to-heavy rainfall and temperatures ≈35°C, can favor NWS fly development (*5*).

Human infestation by *C. hominivorax* causes rapidly progressive and potentially severe myiasis. Clinical manifestations are usually classified according to the involved site as cutaneous, cavitary, or intestinal myiasis (*6*,*7*). Diagnosis relies on identification of larvae, and treatment includes larval removal, wound or lesion care, management of bacterial complications, analgesia, and, in selected cases, antiparasitic drugs such as ivermectin (*8*,*9*).

Mexico was declared free of NWS in 2003; Chiapas was the last state to achieve elimination (*10*,*11*). However, animal cases have been reported in Mexico since 2024 and human cases since 2025, raising concern about reemergence of NWS as a public and animal health problem. Because the same obligate parasite affects livestock, wildlife, companion animals, and humans, animal cases can indicate local circulation of the fly and potential risk for human infestation. In this context, a One Health approach entails coordinated surveillance, diagnosis, reporting, animal movement controls, and fly suppression across human and animal health sectors. Areas along the southern coast of Mexico, including Chiapas, Campeche, Tabasco, and Veracruz, have been considered at increased risk (*12*). A modeling study estimated that the reemerging NWS outbreak was moving northward from Central America at 1.2–1.9 km/day (*13*). We characterized confirmed human NWS myiasis cases in Mexico to provide information for epidemiologic surveillance, prevention, and control.

## Methods

We conducted an observational, descriptive, retrospective study. The study population comprised confirmed human NWS myiasis cases reported in Mexico through the National Epidemiologic Surveillance System from epidemiologic week 16 of 2025 through epidemiologic week 10 of 2026, corresponding to April 13, 2025–March 14, 2026. Those dates represent the epidemiologic reporting period and do not necessarily indicate the dates of infestation onset.

To construct the temporal series, we reviewed official epidemiologic bulletins published by the Directorate General of Epidemiology, Ministry of Health, Mexico, which enabled us to identify the number of confirmed cases reported by epidemiologic week (*14**–**16*). To characterize clinical and epidemiologic features, we used nonidentifiable information available from the official monthly situation reports on human NWS myiasis in Mexico, published by the Directorate General of Epidemiology, Ministry of Health (*17*). We also used cumulative state-level counts from those reports to describe geographic spread at selected epidemiologic reporting weeks; those state-level summaries comprised all 204 confirmed cases.

The unit of analysis for case-level clinical and epidemiologic characterization was each confirmed human NWS myiasis case recorded during the study period, defined as a probable case with taxonomic identification of *C. hominivorax* from a laboratory in the National Network of Public Health Laboratories. We included records with available information on the state and municipality of notification, sex, age, reported anatomic location, concurrent conditions, and discharge or death status. We excluded records with insufficient information for case-level clinical or epidemiologic characterization. Of the 204 confirmed cases included in the official state-level summaries, 2 lacked sufficient case-level information; therefore, we included 202 cases in the clinical and epidemiologic analyses.

We analyzed variables including epidemiologic week of notification, state, municipality, sex, age, reported anatomic location or locations, presence and type of concurrent conditions, number of concurrent conditions, recorded death, and attributed cause of death. The monthly situation reports listed multiple anatomic terms for some patients but did not provide a data dictionary describing how those terms were assigned. When multiple anatomic locations were listed for a patient, we counted each recorded term in the descriptive frequency analysis. We did not interpret multiple listed locations as separate infestations because the reports did not indicate whether they represented distinct infestations, contiguous extension of a single infestation, or different levels of anatomic description. We therefore analyzed reported anatomic locations and concurrent conditions as non–mutually exclusive categories.

We performed descriptive statistical analysis. We summarized categorical variables as absolute frequencies and percentages and age as mean +SD. We did not conduct confirmatory hypothesis testing because of the descriptive design and the absence of a population comparison group. We performed analyses in Microsoft Excel (https://www.microsoft.com) and Stata 17 (StataCorp LLC, https://www.stata.com) software.

This study used nonidentifiable secondary information from public official epidemiologic surveillance sources. The authors did not have access to names, personal identifiers, medical record numbers, addresses, or any information that would permit individual identification. The Local Ethics and Research Committee 703 of the Instituto Mexicano del Seguro Social in Chiapas determined that the study was exempt from formal ethics board review because it was a retrospective secondary analysis of public, nonidentifiable data, involved no intervention, and included no contact with patients. Therefore, informed consent was not required.

## Results

Official state-level surveillance summaries included 204 confirmed human NWS myiasis cases; we used those data to describe cumulative geographic spread during the reporting period. Two records lacked sufficient case-level information for clinical and epidemiologic characterization; therefore, the analyses included 202 cases.

The temporal series derived from the official epidemiologic bulletins included 202 confirmed human NWS myiasis cases. During the 2025 reporting period, 111 cases were recorded, corresponding to a mean of 3 confirmed cases per epidemiologic week. During the first 10 epidemiologic weeks of 2026, a total of 91 confirmed cases were recorded, averaging 9.1 cases per epidemiologic week (Figure 1).

**Figure 1:**
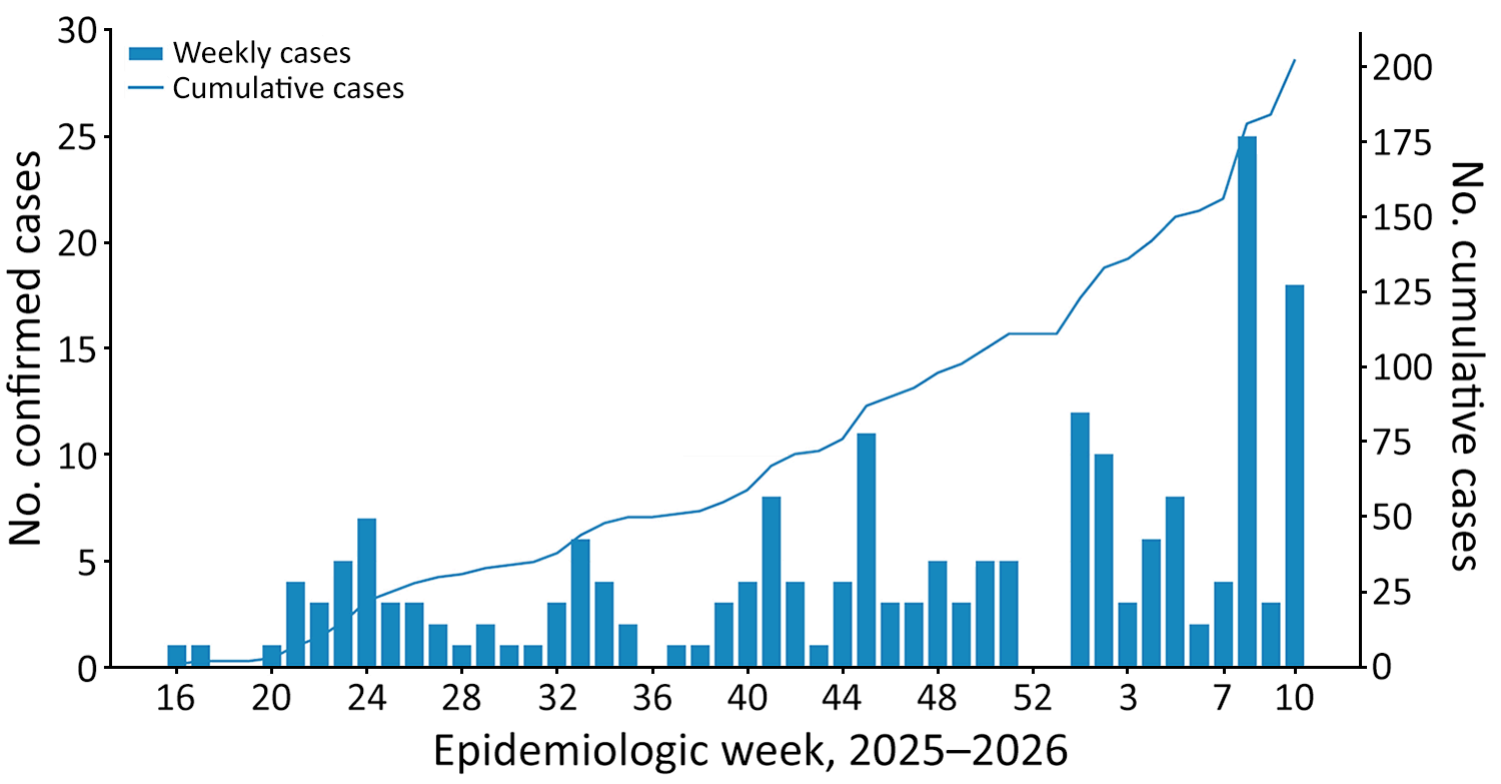
Confirmed human New World screwworm myiasis cases resulting from reemergence of *Cochliomyia hominivorax* screwworm, by epidemiologic week and cumulative, Mexico, April 13, 2025–March 14, 2026. Data were obtained from official epidemiologic bulletins of the Directorate General of Epidemiology, Ministry of Health (*14**–**16*).

The first confirmed cases were reported in Chiapas during epidemiologic week 16 of 2025. Cases were subsequently identified in Campeche in week 20, in Yucatán and Tabasco in week 33, and in Oaxaca in week 47 of the same year. By epidemiologic week 10 of 2026, a total of 12 of Mexico’s 32 states had reported confirmed human NWS myiasis cases.

We mapped the cumulative geographic spread of confirmed cases by state at selected epidemiologic reporting weeks (Figure 2). In the official state-level summaries, Chiapas accounted for 119 (58.3%) of 204 confirmed cases. The next most affected states were Yucatán with 23 (11.3%) cases, Oaxaca with 16 (7.8%) cases, Quintana Roo with 12 (5.9%) cases, Veracruz with 8 (3.9%) cases, Guerrero and Tabasco with 7 (3.4%) cases each, Campeche with 6 (2.9%) cases, Puebla and San Luis Potosí with 2 (1.0%) cases each, and the states of Mexico and Michoacán with 1 (0.5%) case each. The remaining municipality-level, demographic, clinical, and concurrent-condition analyses were based on the 202 records with sufficient case-level information.

**Figure 2:**
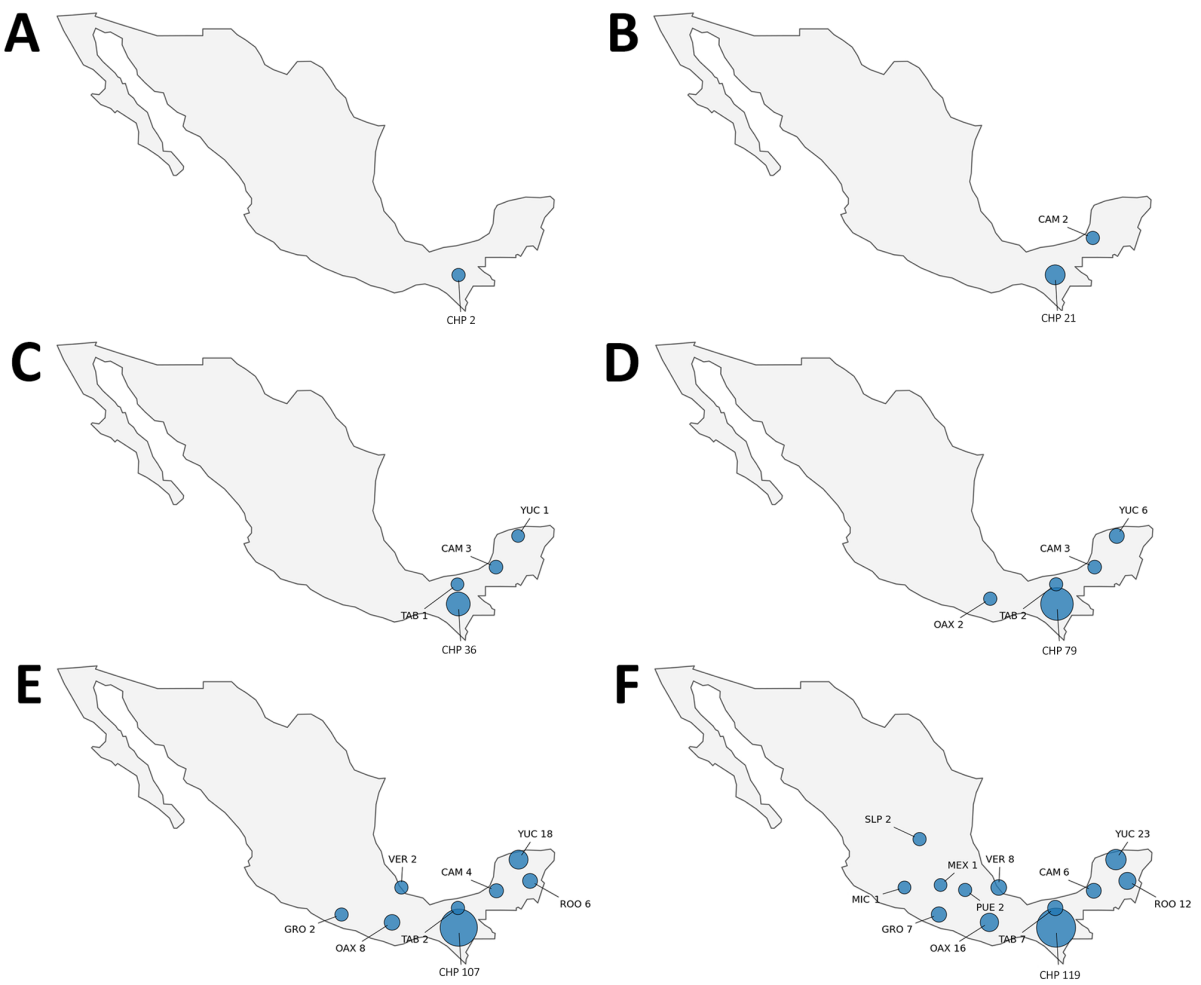
Cumulative geographic spread of confirmed human New World screwworm myiasis cases from reemergence of *Cochliomyia hominivorax* screwworm by state at selected epidemiologic reporting weeks, Mexico, 2025–2026. Panels show cumulative cases reported through each indicated epidemiologic week (EW): A) EW17 of 2025 (n = 2 cases); B) EW24 of 2025 (n = 23 cases); C) EW33 of 2025 (n = 41 cases); D) EW47 of 2025 (n = 92 cases); E) EW5 of 2026 (n = 149 cases); and F) EW10 of 2026 (n = 204 cases). Circle sizes are proportional to the cumulative number of confirmed cases; labels indicate state abbreviation and case count. Counts in this figure reflect all 204 confirmed cases included in official state-level surveillance summaries; 2 records lacking sufficient information were excluded from the individual-level analysis. Data were obtained from official monthly situation reports on human myiasis caused by *Cochliomyia hominivorax* in Mexico, Directorate General of Epidemiology, Ministry of Health (*17*). CAM, Campeche; CHP, Chiapas; GRO, Guerrero; MEX, state of Mexico; MIC, Michoacán; OAX, Oaxaca; PUE, Puebla; ROO, Quintana Roo; SLP, San Luis Potosí; TAB, Tabasco; VER, Veracruz; YUC, Yucatán.

The municipalities with the highest number of cases were Tapachula, Chiapas, with 35 (17.3%) cases; Huixtla, Chiapas, with 14 (6.9%) cases; and Mérida, Yucatán, with 14 (6.9%) cases. Together, those 3 municipalities accounted for 63 cases, representing 31.2% of all cases analyzed.

We summarized the clinical and epidemiologic characteristics of confirmed cases (Table 1). Of the 202 confirmed cases, 145 (71.8%) occurred in male patients and 57 (28.2%) in female patients. Mean age was 61.1 years (SD 19.2, range 12–93 years); for male patients, mean age was 59.2 (SD 19.0) years, and for female patients, 65.9 (SD 18.9) years.

**Table 1:** Clinical and epidemiologic characteristics of confirmed cases of human myiasis resulting from reemergence of *Cochliomyia hominivorax* screwworm, Mexico, April 13, 2025–March 14, 2026*

Characteristic	Male patients, n = 145	Female patients, n = 57	Total, n = 202
Confirmed cases	145 (100.0)	57 (100.0)	202 (100.0)
Age, y			
Mean (SD)	59.2 (19.0)	65.9 (18.9)	61.1 (19.2)
Range	13–90	12–93	12–93
Reported anatomic location			
Lower limbs	66 (45.5)	45 (78.9)	111 (55.0)
Neck	27 (18.6)	14 (24.6)	41 (20.3)
Nose	13 (9.0)	9 (15.8)	22 (10.9)
Mouth	15 (10.3)	6 (10.5)	21 (10.4)
Eyes	10 (6.9)	6 (10.5)	16 (7.9)
Head	10 (6.9)	6 (10.5)	16 (7.9)
Upper limbs	10 (6.9)	4 (7.0)	14 (6.9)
Trunk	9 (6.2)	3 (5.3)	12 (5.9)
Ear	2 (1.4)	3 (5.3)	5 (2.5)
Genitalia	1 (0.7)	1 (1.8)	2 (1.0)
Abdominal cavity	0	2 (3.5)	2 (1.0)
No. concurrent conditions			
0	34 (23.4)	13 (22.8)	47 (23.3)
1	80 (55.2)	27 (47.4)	107 (53.0)
2	20 (13.8)	12 (21.1)	32 (15.8)
3	8 (5.5)	1 (1.8)	9 (4.5)
>4	3 (2.1)	4 (7.0)	7 (3.5)
Concurrent condition			
Diabetes mellitus	29 (20.0)	12 (21.1)	41 (20.3)
Alcoholism	27 (18.6)	0	27 (13.4)
Hypertension	12 (8.3)	14 (24.6)	26 (12.9)
Neoplasia	20 (13.8)	2 (3.5)	22 (10.9)
Conditions associated with cutaneous ulcers	10 (6.9)	5 (8.8)	15 (7.4)
Obesity	5 (3.4)	5 (8.8)	10 (5.0)
Social abandonment/neglect	6 (4.1)	2 (3.5)	8 (4.0)
Altered consciousness	3 (2.1)	4 (7.0)	7 (3.5)
Recent surgery	6 (4.1)	1 (1.8)	7 (3.5)
Venous insufficiency	4 (2.8)	2 (3.5)	6 (3.0)
Other intoxications	4 (2.8)	0	4 (2.0)
Psoriasis	2 (1.4)	1 (1.8)	3 (1.5)
Sensory disorder	1 (0.7)	2 (3.5)	3 (1.5)
Schizophrenia	2 (1.4)	1 (1.8)	3 (1.5)

*Values are no. (%) except as indicated. Percentages for male and female patients were calculated by using sex-specific denominators. Reported anatomic locations and concurrent conditions were not mutually exclusive categories; therefore, percentages may sum to >100%. The source reports did not provide a data dictionary for anatomic location terms or sufficient information to determine whether multiple listed locations represented separate infestations, contiguous extension of a single infestation, or different levels of anatomic description. Concurrent conditions reported in <2 cases are not shown individually. Data source: official monthly situation reports on human myiasis caused by *Cochliomyia hominivorax* in Mexico, Directorate General of Epidemiology, Ministry of Health (*17*).

The most frequent anatomic location for myiasis was the lower limbs, listed in 111 (55.0%) cases, followed by the neck in 41 (20.3%) cases, nose in 22 (10.9%) cases, mouth in 21 (10.4%) cases, eyes and head in 16 (7.9%) cases each, upper limbs in 14 (6.9%) cases, trunk in 12 (5.9%) cases, ear in 5 (2.5%) cases, genitalia in 2 (1.0%) cases, and abdominal cavity in 2 (1.0%) cases. More than 1 anatomic location could be recorded for the same patient. The source reports did not provide sufficient information to determine whether multiple locations represented separate infestations, contiguous involvement from a single infestation, or different levels of anatomic description.

A total of 155 (76.7%) cases had >1 concurrent condition. One concurrent condition was identified in 107 (53.0%) cases, 2 in 32 (15.8%) cases, 3 in 9 (4.5%) cases, and >4 in 7 (3.5%) cases. Overall, 48 (23.8%) cases had >2 concurrent conditions. The most frequently reported concurrent conditions were diabetes mellitus in 41 (20.3%) cases, alcoholism in 27 (13.4%) cases, hypertension in 26 (12.9%) cases, neoplasia in 22 (10.9%) cases, and conditions associated with cutaneous ulcers in 15 (7.4%) cases. The source reports did not consistently provide the type, location, or stage of reported neoplasms. 

During the reporting period, 6 deaths were recorded among patients with confirmed human NWS myiasis (Table 2). At the time of reporting, 1 death was attributed to NWS myiasis. The recorded causes of death for the other 5 patients were basal cell carcinoma, squamous cell carcinoma, encephalitis, respiratory failure, and septic shock. The reports did not provide the interval between diagnosis of NWS myiasis and death, whether the infestation had resolved before death, or sufficient clinical information to assess infestation severity or its potential contribution to the other deaths. The mean age of patients who died was 79.5 (SD 9.6) years. Four (66.7%) deaths occurred in female patients and 2 (33.3%) in male patients. Four (66.7%) deaths were reported in Chiapas, 1 (16.7%) in Yucatán, and 1 (16.7%) in Campeche. The most frequently reported concurrent conditions among patients who died were neoplasia and hypertension, each present in 3 (50.0%) cases, followed by diabetes mellitus and social abandonment/neglect, each present in 2 (33.3%) cases.

**Table 2:** Characteristics of patients with recorded death among confirmed human New World screwworm myiasis cases resulting from reemergence of *Cochliomyia hominivorax* screwworm, Mexico, April 13, 2025–March 14, 2026*

Characteristic	Male patients, n = 2	Female patients, n = 4	Total, n = 6
Cases with recorded death	2	4	6 (100.0)
Age, y			
Mean (SD)	70.5 (13.4)	84.0 (3.6)	79.5 (9.6)
Range	61–80	79–87	61–87
State			
Chiapas	2	2	4 (66.7)
Yucatán	0	1	1 (16.7)
Campeche	0	1	1 (16.7)
Recorded cause of death			
New World screwworm myiasis	0	1	1 (16.7)
Basal cell carcinoma	0	1	1 (16.7)
Squamous cell carcinoma	0	1	1 (16.7)
Encephalitis	1	0	1 (16.7)
Respiratory failure	0	1	1 (16.7)
Septic shock	1	0	1 (16.7)
No. concurrent conditions			
1	1	0	1 (16.7)
2	1	2	3 (50.0)
4	0	1	1 (16.7)
5	0	1	1 (16.7)
Concurrent condition			
Neoplasia	1	2	3 (50.0)
Hypertension	1	2	3 (50.0)
Diabetes mellitus	1	1	2 (33.3)
Social abandonment/neglect	0	2	2 (33.3)
Altered consciousness	0	1	1 (16.7)
Conditions associated with cutaneous ulcers	0	1	1 (16.7)
Sensory disorders	0	1	1 (16.7)
Immobility	0	1	1 (16.7)
Wasting syndrome	0	1	1 (16.7)
Chronic kidney disease	0	1	1 (16.7)

*Values are absolute no. or no. (%) except as indicated. Recorded causes of death reflect the classifications provided in the official source reports. The reports did not provide the interval between diagnosis of New World screwworm myiasis and death, whether the infestation had resolved before death, or sufficient information to assess infestation severity or its contribution to deaths attributed to other causes. Concurrent conditions were not mutually exclusive categories; therefore, percentages may sum to >100%. Data source: official monthly situation reports on human myiasis caused by *Cochliomyia hominivorax* in Mexico, Directorate General of Epidemiology, Ministry of Health (*17*).

## Discussion

In this national series of confirmed human NWS myiasis cases in Mexico, the main findings were concentration of reported cases in southern Mexico, particularly Chiapas, as well as a higher mean number of weekly confirmed cases during the first 10 epidemiologic weeks of 2026 than during the 2025 reporting period, predominance among adult men, frequent reporting of lower-limb involvement, and a high proportion of patients with concurrent clinical or social conditions. Six deaths were reported among patients with NWS myiasis, and at the time of reporting, 1 death was attributed to NWS myiasis. Those findings provide early information about the clinical and epidemiologic characteristics of human infestation during the reemergence of NWS in Mexico.

The concentration of cases in Chiapas, particularly in municipalities such as Tapachula and Huixtla, is consistent with the initial distribution observed in southern Mexico. That distribution overlaps geographically with areas reporting high numbers of NWS myiasis cases in animals, according to reports from the National Service for Agrifood Health, Safety, and Quality (*18*). However, that overlap should be interpreted with caution because our study did not assess the individual-level or ecologic relationships between animal and human cases. Even so, the geographic overlap highlights the need to strengthen integrated surveillance among human health, animal health, and animal movement control systems, particularly in border, livestock-producing, and environmentally suitable areas where NWS could become established or disperse (*19*,*20*).

The higher mean number of weekly confirmed cases during the first epidemiologic weeks of 2026, compared with the 2025 reporting period, could reflect a combination of territorial spread, increased clinical detection, strengthened reporting, or a true increase in transmission. Because this study was descriptive and based on secondary surveillance information, we could not distinguish among those mechanisms. Nevertheless, the observed temporal pattern underscores the need to maintain active epidemiologic surveillance, improve clinical diagnostic capacity, and ensure timely notification of suspected and confirmed cases.

The predominance of cases among adult men is consistent with findings from other series of human myiasis, in which higher frequencies have been reported among male patients (*21*,*22*). That pattern could be related to differences in occupational exposure, outdoor activities, contact with animals, unattended wounds, or delayed healthcare seeking. However, those variables were not available in the data sources we used, and therefore, we could not assess them directly. The older mean age of patients also suggests that adults and older persons with concurrent clinical conditions might represent a particularly vulnerable group.

The lower limbs were the most frequently reported anatomic location, consistent with previous studies reporting frequent involvement of the legs and feet (*23**–**25*). Wounds, chronic ulcers, trauma, and other cutaneous lesions in those anatomic regions might increase the risk for infestation, particularly among persons with diabetes mellitus, venous insufficiency, sensory disorders, or other conditions that can delay recognition of skin lesions. However, the surveillance reports did not provide sufficient information for us to determine whether multiple recorded anatomic terms represented separate infestations or contiguous extension of a single infestation. In that context, wound and lesion care, periodic examination of feet and lower limbs, and intentional examination for larvae in patients with exposed lesions remain clinically relevant.

The high proportion of cases with concurrent conditions highlights that NWS myiasis should be considered not only as an acute parasitic infestation but also as a condition favored by clinical and social vulnerability (*26**–**29*). In this series, the most frequent concurrent conditions were diabetes mellitus, alcoholism, hypertension, neoplasia, and conditions associated with cutaneous ulcers. Those findings suggest that chronic diseases, persistent skin lesions, sensory disorders, immobility, social abandonment or neglect, and other conditions that impair self care could contribute to the clinical manifestation of infestation. However, because this study was a case series without a comparison group, we could not estimate the magnitude of risk associated with each concurrent condition.

Six deaths were recorded among patients with confirmed human NWS myiasis; however, only 1 was attributed to NWS myiasis at the time of reporting. The official reports did not provide the interval between diagnosis of infestation and death, whether the NWS infestation had resolved before death, or detailed information about the number of larvae, extent of tissue involvement, treatment, secondary infection, or other indicators of infestation severity. Consequently, we could not assess the contribution of NWS myiasis to the other reported deaths. Nevertheless, the occurrence of deaths among older patients with substantial concurrent clinical and social vulnerability underscores the importance of timely detection, larval removal, lesion care, management of secondary bacterial infection, and comprehensive treatment of underlying conditions.

The first limitation of our study is that it was based on epidemiologic surveillance information and, therefore, depends on the quality, timeliness, consistency, and completeness of reporting. Second, underreporting of human NWS myiasis is possible, particularly for cases that were not clinically recognized, taxonomically confirmed, or reported through the national surveillance system. The available information did not permit classification of cases by severity or number of larvae. Third, population denominators were unavailable, so we could not estimate incidence, population-based rates, or individual risk. Fourth, the source reports did not provide a data dictionary for anatomic location terms, so we could not determine whether multiple recorded locations represented separate infestations, contiguous extension of a single infestation, or different levels of anatomic description. Head and neck designations and combinations of adjacent structures, such as the nose, mouth, and eye, were particularly difficult to interpret. Fifth, available clinical information did not permit analysis of occupation, animal contact, wound or lesion type, time from symptom onset, treatment received, duration of infestation, resolution, or subsequent clinical course. The type, location, and stage of neoplasia were not consistently reported. For patients with recorded death, the reports did not provide the interval from NWS infestation to death, whether the infestation had resolved, or sufficient information to determine infestation severity or contribution to death. Finally, because of the descriptive design and absence of a comparison group, our findings cannot establish causal associations between concurrent conditions, animal exposure, environmental conditions, and the occurrence of human NWS myiasis.

The reemergence of NWS in Mexico and the resulting human NWS myiasis cases represent an event of public and animal health importance. The initial concentration of reported human cases in southern Mexico, predominance among adult men, frequent reporting of lower limb involvement, and high proportion of patients with concurrent conditions support strengthened clinical detection, timely reporting, and integrated One Health surveillance. Earlier diagnosis and treatment could help prevent progressive tissue destruction, secondary bacterial infection, and functional impairment.

Because animal and human cases reflect northward spread of the NWS fly and that spread is expected to continue, surveillance, diagnostic preparedness, animal movement controls, and fly-suppression activities should be implemented proactively at the leading edge of expansion, rather than only after human cases are detected. Coordination between human health, animal health, wildlife, and agricultural sectors will be essential to guide prevention and control measures.
